# KRAS-related miR-143 expression is associated with lymph node involvement and correlates with outcome in pancreatic adenocarcinoma patients

**DOI:** 10.3389/fonc.2023.1295936

**Published:** 2023-12-05

**Authors:** Daniele Lavacchi, Simone Polvani, Antonio Taddei, Federico Scolari, Luca Messerini, Enrico Caliman, Luca Moraldi, Alessia Guidolin, Gian Luca Grazi, Andrea Galli, Serena Pillozzi, Lorenzo Antonuzzo

**Affiliations:** ^1^ Clinical Oncology Unit, Careggi University Hospital, Florence, Italy; ^2^ Department of Clinical and Experimental Biomedical Sciences “Mario Serio”, University of Florence, Florence, Italy; ^3^ HPB Surgery Unit, Careggi University Hospital, Florence, Italy; ^4^ Department of Experimental and Clinical Medicine, University of Florence, Florence, Italy; ^5^ Department of Health Sciences, University of Florence, Florence, Italy

**Keywords:** pancreatic cancer, miRNA, KRAS, miR-143, miR-155, miR-206

## Abstract

**Introduction:**

Pancreatic adenocarcinoma (PC) is one of the most lethal malignancies; even after resection the patients’ 5-year disease-free survival (DFS) is lower than 26%. The genetic mutational landscape of PC is dominated by activating KRAS mutations, that have been reported in approximately 90% of cases; however, beyond KRAS - direct mutations, several KRAS-targeting miRNAs appear to be downregulated, strengthening the already activated RAS signaling. In addition, the interplay between miRNAs and RAS includes poorly investigated downstream miRNAs. The aim of this study was to determine the prognostic value of some of these candidate KRAS-related miRNAs.

**Patients and methods:**

Between 2015 and 2022, 44 patients with pathologically confirmed PC, who received surgery and were enrolled by the Clinical Oncology Unit, Careggi University Hospital, Florence (Italy). PC Total RNA was extracted from FFPE sections, retro-transcribed and the resulting cDNA was then used for qPCR analysis. A panel of KRAS-related miRNA (miR-155, miR-206 and miR-143) was analyzed.

**Results:**

In this observational study patients sex distribution was unequal with 34.1% being male and 65.9% female. The most frequent tumor localization was the head of the pancreas (65.9%) and the pathological stages were pT1-2 (45.5%), pT3 (54.5%), pN0 (22.7%), pN+ (77.3%). Adjuvant therapy was administered to 63.6% of patients; disease recurrence was observed in 69% of cases. Twenty-three patients, whose RNA was of adequate quality, were used in the mRNAs expression studies. When comparing the miRNA expression between PC and a pool of healthy tissues, miR-155 was overexpressed and miR-206 downregulated in PC, while miR-143 expression was unchanged. However, when categorized in low- and high- miR-143 expressing PC (according to the median value), high miR-143 was associated with nodal involvement (pN+) (p=0.029), who in turn was linked with shorter DFS (p=0.009) and overall survival (OS) (p=0.021) compared to pN0. A trend toward inferior DFS was observed for higher expression of miR-206 (p=0.095) and miR-143 (p=0.092). Finally, responders to a first-line treatment for advanced disease had miR-155 overexpressed (p=0.048).

**Conclusions:**

miRNAs are involved in PC tumorigenesis and metastatic spread. In light of miR-143 association with lymphatic spread and poor prognosis, a comprehensive analysis of miRNA interplay with KRAS deserves further investigation.

## 1 Introduction

Pancreatic adenocarcinoma (PC) is one of the most lethal tumors accounting for more than 400,000 deaths worldwide annually; given the poor prognosis, incidence and mortality still have similar rates (1–3). Five-year overall survival (OS) is less than 10%. This dismal prognosis is mainly due to the late diagnosis in over 80% of cases and biological aggressiveness even in patients who are candidates for surgical resection (4). Despite adjuvant treatments, 5-year disease-free survival (DFS) rate remains approximately 19-26%. Multivariable analysis from the PRODIGE 24 trial showed that mFOLFIRINOX, age, grade, stage, and larger-volume center were significantly associated with OS (5). To date, no molecular determinant has been reported to confer a favorable prognostic profile in patients who underwent surgical resection for PC.

In large international cohorts of patients, genomic profile analyses showed that KRAS mutations are reported in approximately 90% of PC cases (6). Among KRAS-activating mutations, G12C KRAS is the first one successfully targeted but represents accounts for only 1-2% of mutated KRAS cases. Preliminary data from the ongoing phase I/II CodeBreaK100 trial (NCT03600883) demonstrated a remarkable activity of the covalent KRAS-G12C inhibitor sotorasib in heavily pretreated patients with G12C-mutated PC (7). Similar encouraging results have been reported for adagrasib in the phase I/II KRYSTAL-1 trial (NCT03785249) (8). The remaining 10% of PCs have no oncogenic driver mutations in KRAS, but may harbor several other molecular alterations, including TP53 and BRAF mutations, microsatellite instability (MSI), gene fusions/amplifications (e.g. BRAF, NTRK, FGFR1-3, ALK, RET, NRG1, ERBB2, MET), and alterations in DNA-damage repair genes (e.g. BRCA2, ATM, PALB2, ARID1A, PBRM1, SMARCA4) or regulators of cell-cycle progression (e.g. CDKN2A, CCND1, CCNE1) (6, 9).

However, the molecular features characterizing the progression and chemosensitivity of these neoplasms are largely unknown. Beyond PC gene expression signatures, the study of genetic and epigenetic mechanisms regulating the multistep processes involved in transcription or translation might unravel novel prognostic factors. In recent years, microRNAs (miRNAs) have emerged as a critical class of negative regulators of gene expression, through modulation of post-transcriptional activity of multiple target mRNAs, suggesting their role as putative predictors of prognosis or treatment response (10–13).

It is noteworthy that despite the activation of KRAS through gene mutation in the vast majority of cases, several miRNAs capable of directly targeting KRAS, including miR-96, miR-126, and miR-217, are simultaneously downregulated (12–15). Yu et al. and Zhao et al. showed that upregulation of miR-96 and miR-217, respectively, led to the reduced level of KRAS protein and, consequently, constitutive activation of the AKT pathway (13, 14). Since the reduced expression of these miRNAs correlates with increased expression of KRAS, it is likely that these alterations represent a mechanism for strengthening the already activated RAS signaling. Several miRNAs that were proven to target and inhibit the expression of RAS oncoproteins, are generally downregulated in PC, thus concurring with reciprocal overexpression and activation of RAS, irrespective of activating gene mutations (16). The interplay between miRNAs and RAS is not only a consequence of miRNAs acting as negative modulators of RAS, but also includes downstream miRNA effectors. A crucial role is undoubtedly played by miR-21, which is up-regulated by KRAS oncogenic mutants in PC as well as many other human cancers (17).

The aim of this study was to characterize the expression of candidate KRAS-related miRNAs in a cohort of pancreatic specimens and to correlate these data with clinical outcomes.

## 2 Materials and methods

### 2.1 Patients and biopsy processing

In this single-center observational prospective study we collected surgical specimens for miRNA analysis and clinical-demographic data of patients with pathologically confirmed PC, who underwent surgical resection for operable disease and referred to the Clinical Oncology Unit, Careggi University Hospital, Florence (Italy) between 2015 and 2022. All patients enrolled in the study received radical surgery upfront after assessment of resectability by a multidisciplinary team. Clinical stage was assessed according to clinical practice, routinely with CT scan, US-endoscopy and, whenever appropriate, MRI and/or FDG-PET scan. All participants gave written informed consent before enrolment. Inclusion criteria also required at least 18 years of age and written informed consent. Patients were excluded if they had metastatic or locally advanced inoperable disease or other type of neoplasms. All adjuvant treatments performed according to clinical practice were admitted. All treatments were performed at the investigator’s discretion according to the observational nature of the study.

Pancreatic specimens were immediately fixed in 4% formaldehyde (FF) for 24h. After three washings in PBS (3 hours each) the specimens were transferred in 50 ml sterile tubes containing a sterile solution of 15% sucrose in PBS. Specimens were stored at 4°C in this solution until sinked to the bottom of the tube; next, sample tissues were transferred to a 30% sterile sucrose solution, again until sinked to the bottom. Before embedding in OCT, the tissues were blotted on whatman paper to remove excess sucrose then stored at -80°C.

Archival FF tumor samples of patients enrolled were used for the analysis of a panel of KRAS-related miRNA, namely miR-155, miR-206 and miR-143. Molecular data on mi-RNAs were available for a cohort of n=25 samples. Six pancreas tissue samples from healthy donors were used as the control group for the mi-RNAs analysis.

### 2.2 RNA extraction and quantitative real-time reverse transcription PCR

Total RNA was extracted from FF cryosections using the RNeasy Mini kit (Qiagen, Manchester, UK), with minor modifications; specifically, four to five 25 μm thick cryo-sections were transferred to 1.5ml microcentrifuge tube and washed two times with molecular biology grade water to remove the OCT medium. After the second wash the supernatant was removed and the sections processed according to the kit protocol. The quantity and the purity of RNA were evaluated using a Nanodrop spectrophotometer. RNA from each sample was retro-transcribed using miRCURY LNA RT kit (Qiagen, Manchester, UK); qPCR analysis was performed on a Rotor Gene Q (Qiagen, Manchester, UK) using miRCURY LNA SYBER Green PCR kit (Qiagen, Manchester, UK). The primers used were miRCURY LNA miRNA PCR Assays primer sets: hsa-miR-143-5p (catalog number YP00204570), hsa-miR-155-5p (catalog number YP02119311), hsa-miR-206 (catalog number YP00206073), hsa-miR-103a-3p (103a-3p Assay). The relative quantification was performed using Rotor-Gene Q Series Software (version 2.3.5) and the data were normalized to miR-103a-3p.

### 2.3 The Cancer Genome Atlas analysis

miRNA expression and clinical data were extracted from The Cancer Genome Atlas TCGA dataset comprising 178 tissue samples of pancreatic adenocarcinoma. Data retrieval was carried out using the TCGAbiolink R package (version 2.28.4). To ensure data quality, expression data has been filtered, removing miRNAs with counts lower than 10 in the majority of samples. Subsequently, the miRNA counts were z-score normalized.

### 2.4 Statistical analysis

Patient characteristics were presented by descriptive statistics, using median and range for continuous variables and number and percentage for categorical variables. Statistical analysis was performed using RStudio (18). Relative expression levels were calculated using the ΔΔCt method (19), with miRNA miR-103 as the reference and the geometric mean of expression levels in 6 healthy tissue samples as the negative control. Correlations between miRNA relative expression and clinical parameters were assessed using the non-parametric Wilcoxon rank-sum test, after observing the non-normal distribution of the data. To categorize miRNA relative expression as high or low, we employed the median value as a cutoff point, enabling the differentiation of patients with differing miRNA expression profiles. In cases where multiple testing corrections were required, we applied the Bonferroni method to maintain the significance threshold. Kaplan-Meier curves were generated using the Survival package (20), illustrating the correlation between miRNA expression levels or clinical parameters and OS or PFS. Multivariate analysis was carried out using the Cox regression technique to further explore the relationship between miRNA expression and survival outcomes while controlling for potential confounding factors.

### 2.5 Ethics and regulatory considerations

The present study was approved by the Regional Ethics Committee for Clinical Trials of the Tuscany Region (Firenze, Italy; no. 0028114 - 2014). All informed consent documents were in compliance with the International Conference on Harmonization (ICH) guideline on good clinical practice (GCP). The study protocol was performed in accordance with the principles of the Declaration of Helsinki and in compliance with GCP and the applicable laws and regulations. Each patient was identified by a code instead of the patient’s name in order to protect the patient’s identity when reporting study-related data.

## 3 Results

### 3.1 Patients characteristics

Forty-four patients with PC who underwent upfront surgical resection for operable disease during the study period were included in the analysis. The clinical baseline characteristics of the enrolled patients are summarized in **Table 1**. The median age at the time of diagnosis was 75 years (range 55-91 years), 15 were males (34.1%) and 29 were females (65.9%). At enrolling time, the ECOG PS was 0 in 24 patients (54.5%), 1 in 11 patients (25%) while it was not reported for 9 patients (20.5%). Tumor was located in the pancreatic head in 30 patients (68.2%) and in the body-tail of pancreas in 14 patients (31.8%) and 19 patients (43.2%) had jaundice at diagnosis. Clinical staging (cTNM) was reported in detail in **Table 1**. Forty-three patients had a resectable tumor at diagnosis, while 1 patient was classified as borderline resectable. Type of surgical intervention was duodenocephalopancreasectomy (DCP) for most of the patients (n=29, 65.8%) and all but 1 patient achieved R0 surgery. The histological diagnosis was ductal adenocarcinoma for the entire study population (n=44) and the tumor grading was G2 (grade 2, moderately differentiated) in 39 patients (88.6%) and G3 (poorly differentiated) in 5 patients (11.4%). Pathological staging according to the VIII edition of TNM staging system was as follows: pT1 in 5 patients (11.4%), pT2 in 15 patients (34.1%), pT3 in 24 patients (54.5%); the lymph nodes involvement was pN0 for 10 patients (22.7%), pN1 for 28 patients (63.7%) and pN2 for 6 patients (13.6%). Most patients (n=28, 63.6%) had pathological stage IIB, 6 patients (13.6%) stage IIA and 6 (13.6%) stage III. According to the pathological stage and clinical conditions, 28 patients (63.6%) received adjuvant treatment with either gemcitabine, mFOLFIRNOX or capecitabine. Median follow-up was 17 months (Inter-Quartile Range 8-30 months). Twenty-nine patients (65.9%) experienced disease recurrence. Of these, 19 patients (65.5%) received systemic chemotherapy as first-line treatment. Twenty-eight patients (63.6%) were dead at the cut-off date for the follow-up analysis, while 16 patients (36.4%) were alive and undergoing systemic treatment or follow up as indicated per clinical practice.

**Table 1:** Patients’ baseline characteristics.

Patients (n = 44)	
Age	
Median (range) – years	75 (55-91)
Sex – no. (%)	
Male	15 (34.1)
Female	29 (65.9)
ECOG Performance Status – no. (%)	
0	24 (54.5)
1	11 (25)
Unknown	9 (20.5)
Histotype and tumor grading - no. (%)	
Ductal adenocarcinoma	44 (100)
Grade 1	0 (0)
Grade 2	39 (88.6)
Grade 3	5 (11.4)
Tumor location - no. (%)	
Head	30 (68.2)
Body-Tail	14 (31.8)
Tumor at diagnosis - no. (%)	
cT1	3 (6.8)
cT2	21 (47.7)
cT3	19 (43.2)
Unknown	1 (2.3)
Clinical lymph nodes involvement – no. (%)	
cN0	18 (40.9)
cN1	17 (38.6)
cN2	9 (20.5)
Type of surgery – no. (%)	
duodenocephalopancreasectomy	29 (65.8)
splenopancreatectomy	12 (27.3)
total pancreatectomy	3 (6.9)
Pathological staging (pTNM) - no. (%)	
IA	2 (4.5)
IB	2 (4.5)
IIA	6 (13.6)
IIB	28 (63.6)
III	6 (13.6)
Adjuvant therapy – no. (%)	
No	16 (36.4)
Yes	28 (63.6)
Gemcitabine	17 (38.6)
mFOLFIRINOX	10 (22.7)
Capecitabine	1 (2.3)
Disease recurrence - no. (%)	
No	15 (34.1)
Yes	29 (65.9)
Death - no. (%)	
No	16 (36.4)
Yes	28 (63.6)

### 3.2 Expression of miRNAs panel and their association with clinicopathological characteristics

**Figure 1:**
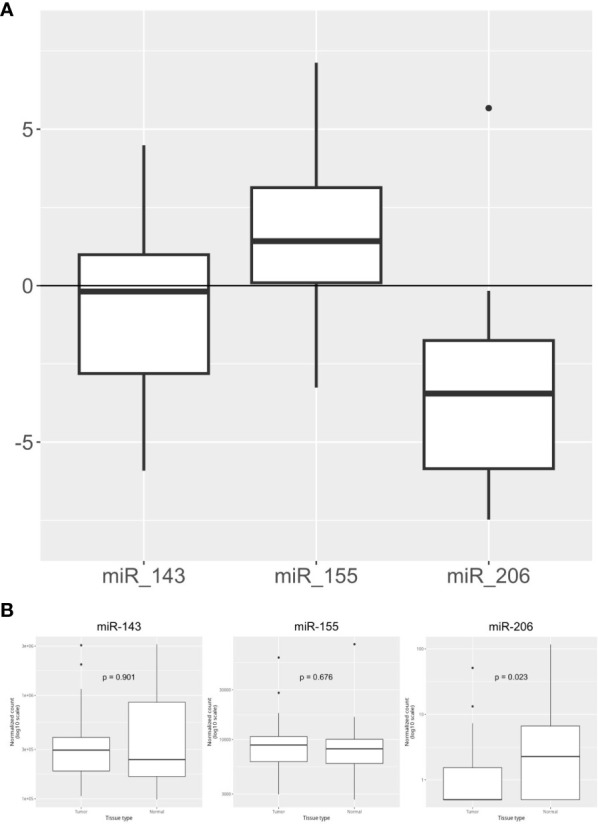
Overall expression of miR-143, mi-R155, and miR-206. **(A)** miRNA relative expression levels obtained in our PC cohort (n=44) with ΔΔCt method on qPCR results. Expression values have been log-transformed. miRNA expression in healthy tissue samples is represented by the 0 baseline. **(B)** miRNA expression levels in Tumor vs Adjacent Normal Tissue samples obtained from TCGA public datasets (n=84 samples).

The expression levels of the three KRAS-related miRNAs (i.e. miR-155, miR-206 and miR-143) were obtained from diagnostic FFPE tumor samples. Compared to healthy controls, tumor samples showed a higher level of miR-155 and a lower level of miR-206, while miR-143 has a similar expression in tumors and in healthy tissues. In detail, the median expression values of the studied miRNAs are shown in **Figure 1A**. Expression of miR-155, miR-206 and miR-143 was also evaluated on data obtained from online databases. RNA-Seq reads were retrieved from TCGA database and a pairwise comparison was performed on miRNA expression levels in 42 PC tumor samples vs 42 PC adjacent normal tissue samples (**Figure 1B**). We then assessed whether miRNAs expression levels correlated with the main clinicopathological features of the study population, such as sex, age (< or ≥70 years), tumor stage and grade and response to therapy. A statistically significant correlation was found between high expression of miR-155 and female sex (p=0.03) and between high expression of miR-155 and response to first-line chemotherapy (p=0.047). Moreover, high miR-143 expression showed a significant correlation with lymph node involvement in the surgical samples (p=0.029). On the other hand, no statistical association between miR-206 expression and the main characteristics of patients was found (**Table 2**).

**Table 2:** Correlation between miRNA expression and clinical data. Bold = P<0.05.

		miR_155		miR_143		miR_206	
		mean expression	P	mean expression	p	mean expression	p
Sex	M	7,8 (0,104 - 54,2)	**0,031**	3,51 (0,0004 - 22,4)	0,935	0,226 (0,009 - 0,723)	0,196
	F	17,4 (0,316 - 140)		1,59 (0,017 - 7,10)		3,55 (0,004 - 51)	
Age	Under 70	19,1 (0,104 - 140)	0,650	1,85 (0,0004 - 7,30)	0,852	4,43 (0,006 - 51)	0,295
	Over 70	8,4 (0,131 - 54,2)		2,82 (0,017 - 22,4)		0,176 (0,004 - 0,891)	
Grading	II	14,2 (0,104 - 140)	0,497	2,61 (0,0004 - 22,4)	0,446	2,50 (0,004 - 51)	0,844
	III	8,35 (2,17 - 15,8)		0,475 (0,113 - 0,828)		0,163 (0,021 - 0,436)	
Site	Head	3,81 (0,104 - 18)	0,138	2,90 (0,017 - 22,4)	1	3,53 (0,004 - 51)	0,379
	Body-Tail	28,6 (0,280 - 140)		1,70 (0,0004 - 7,30)		0,174 (0,006 - 0,436)	
Best Response	SD + PR + CR	37 (0,280 - 140)	**0,048**	1,33 (0,0004 - 3,33)	0,530	0,114 (0,006 - 0,258)	0,876
	PD	0,996 (0,104 - 2,20)		5,26 (0,019 - 22,4)		0,199 (0,007 - 0,723)	
pN	N_0	2,44 (0,280 - 7,02)	0,243	0,219 (0,0004 - 0,699)	**0,029**	0,039 (0,012 - 0,118)	0,243
	N+	16,3 (0,104 - 140)		2,89 (0,017 - 22,4)		2,76 (0,004 - 51)	
Adjuvant therapy	No	8,06 (0,131 - 54,2)	0,495	3,42 (0,017 - 22,4)	0,935	0,122 (0,004 - 0,723)	0,081
	Yes	17,2 (0,104 - 140)		1,64 (0,0004 - 7,30)		3,62 (0,006 -51)	

Reported values are 2^-(ΔΔCq)^, normalized to control healthy pancreatic tissues.

bold= P<0.05.

### 3.3 Survival outcomes analysis

In the overall population, our results showed a median DFS (mDFS) of 11 months (95% CI, 10-19) and a median OS (mOS) of 23 months (95% CI, 17-45).

#### 3.3.1 Survival analysis according to the clinical characteristics of patients

**Figure 2:**
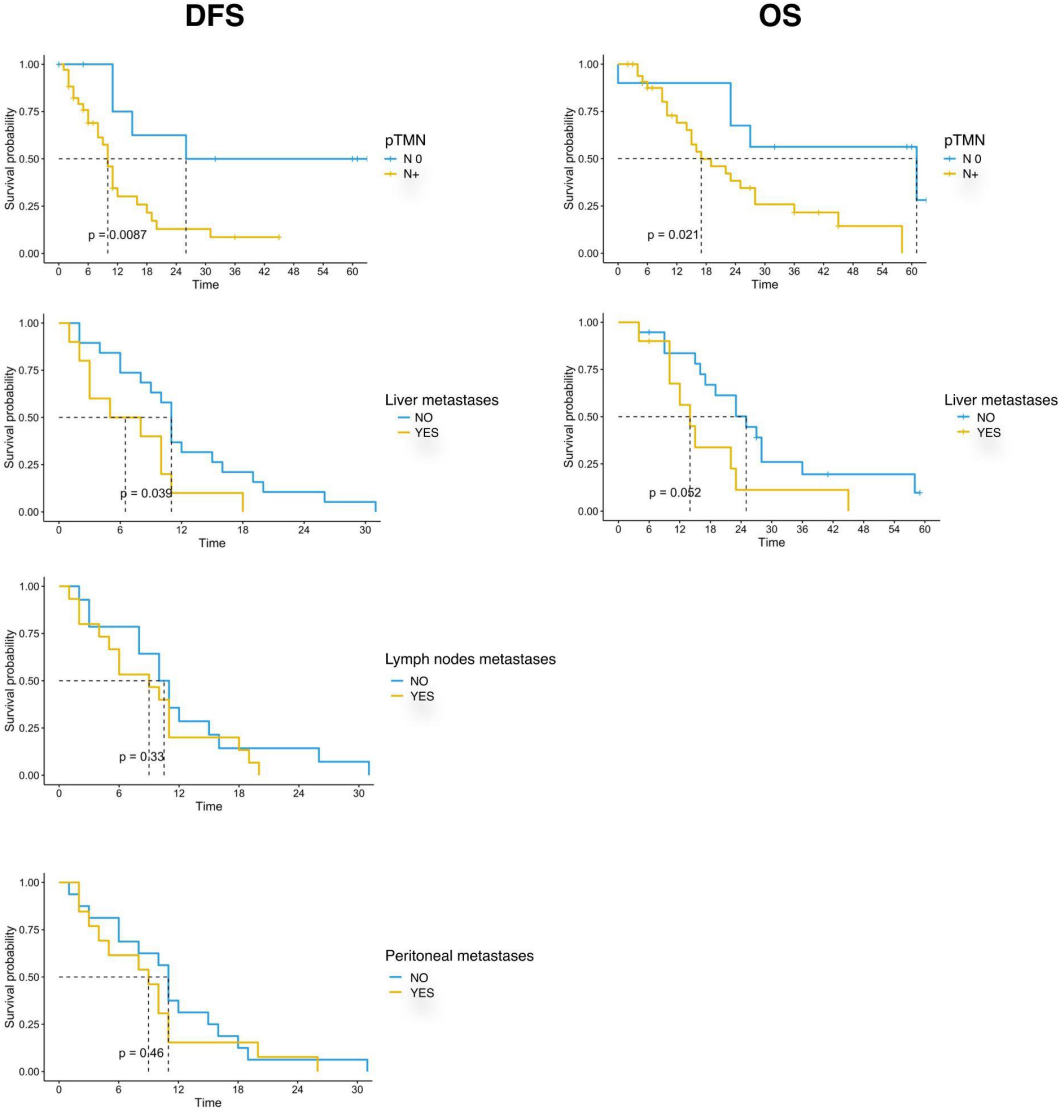
Kaplan-Meier curves for DFS and OS by clinical and pathological characteristics (pTMN N0 vs N+; Liver metastases; Lymph nodes metastases; Peritoneal metastases) of PC patients (n=44) enrolled in the study. Significance levels have been computed with log-rank tests.

Our data showed that patients with lymph node involvement at pathological diagnosis had significantly poorer survival. mDFS was 26 months (95% CI, 15-NR) in N0 patients and 10 months (95% CI, 8-16) in N+ patients (p=0.005) while mOS was 61 months (95% CI, 27-NR) versus 17 months (95% CI, 14-36) in N0 and N+ patients, respectively (p=0.003). Furthermore, we found that the location of metastases at the time of disease recurrence was associated with survival. Patients with liver metastases had a significantly shorter DFS and OS compared with those with other sites of metastasis. In detail, mDFS was 12 months (95% CI 11-31 months) versus 9 months (95% CI 5-18 months), p=0.039, and mOS was 27 months (95% CI, 19-NR) versus 14 months (95% CI, 10-NR), p=0.027. Moreover, recurrence in the lymph node and peritoneum was associated with a significantly worse DFS (p=0.01 for recurrence in the lymph node and p=0.0192 for recurrence in the peritoneum, respectively) and with a trend for a worse OS (**Table 3**; **Figure 2**).

**Table 3:** Kaplan-Meier estimates of survival.

Category		DFS		OS	
		Median (months)	p	Median (months)	p
Sex	M	15 (10 – NA)	0,511	23 (22 – NA)	0,752
	F	11 (8 – 31)		19 (14 – NA)	
Age	Under 70	11 (10 – 26)	0,527	25 (17 – NA)	0,751
	Over 70	12 (9 – NA)		23 (10 – NA)	
Site	Head	11 (9 – 26)	0,881	23 (15 – NA)	0,555
	Body-Tail	10 (8 – 16)		36 (15 – NA)	
pN	N_0	26 (15 – NA)	**0,0056**	61 (27 – NA)	**0,0037**
	N+	10 (8 – 16)		17 (14 – 36)	
Liver metastases	No	12 (11 – 31)	**0,0039**	27 (19 – NA)	**0,027**
	Yes	9 (5 – 18)		14 (10 – NA)	
Lymph nodes metastases	No	12 (10 – NA)	**0,01**	23 (17 – NA)	0,349
	Yes	9 (5 – 18)		22 (14 – NA)	
Local metastases	No	11 (10 – 20)	0,238	23 (15 – 45)	0,980
	Yes	8 (2 – NA)		27 (10 – NA)	
Peritoneal metastases	No	12 (11 – NA)	**0,0192**	23 (19 – NA)	0,136
	Yes	9 (4 – NA)		17 (10 – NA)	
Lung metastases	No	11 (10 – 20)	0,309	23 (16 – 36)	0,815
	Yes	6 (5 – NA)		30 (10 – NA)	

bold= P<0.05.

#### 3.3.2 Survival analysis according to miRNA expression

**Figure 3:**
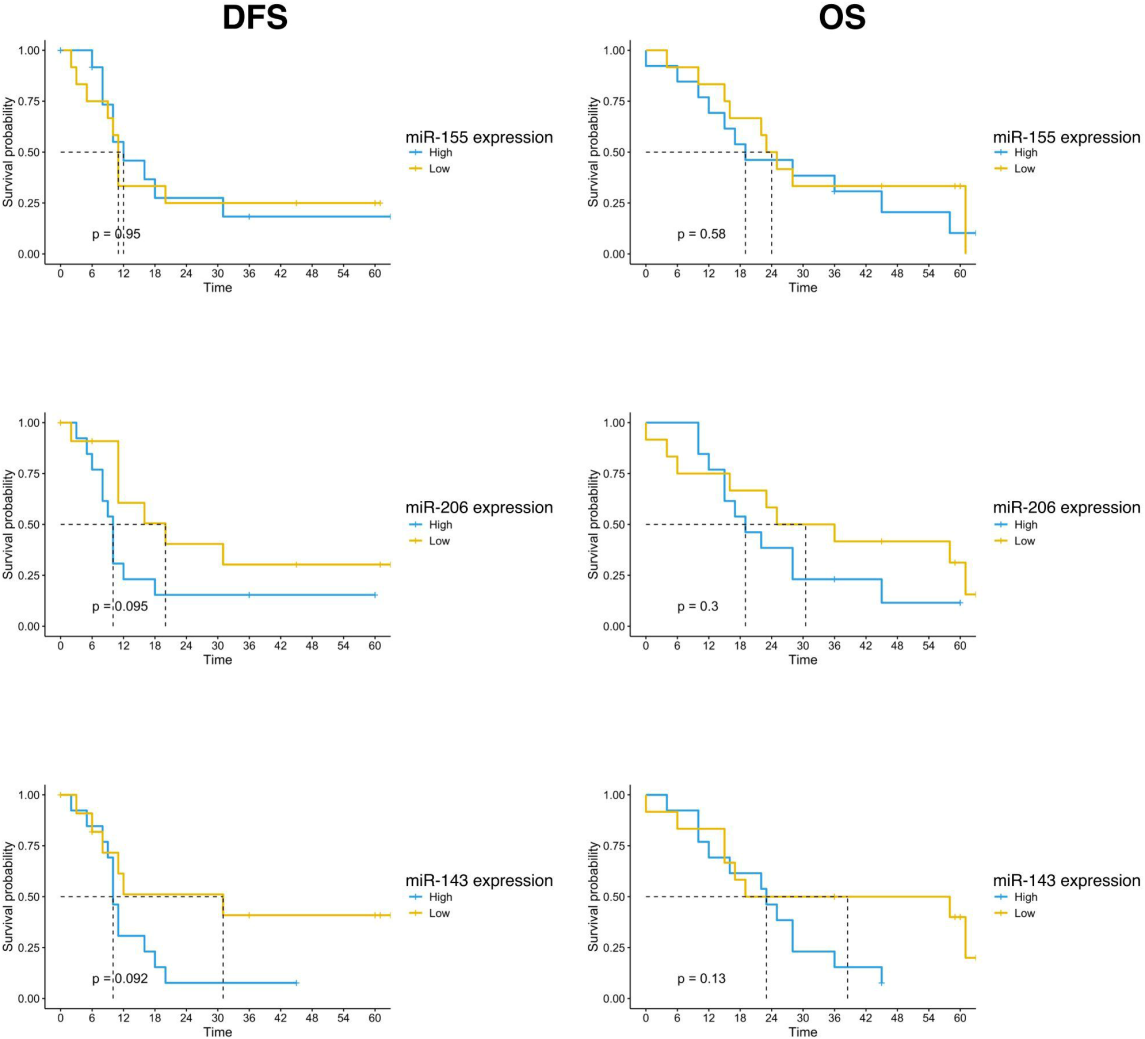
Kaplan-Meier curves for DFS and OS by miR-155, miR-206 and miR-143 expression of PC patients (n=25) enrolled in the study. Significance levels have been computed with log-rank tests.

Survival outcomes analysis was also performed according to the expression of the three studied miRNAs. The population was divided into high and low expression groups, based on the median expression value of each miRNA. Patients with low miR-143 expression in the tumor sample showed a trend of better survival than patients with high miRNA-143 expression. The mDFS was 31 months (95% CI, 8-NR) versus 10 months (95% CI, 9-NR) (p=0.092) and the mOS was 58 months (95% CI, 17-NR) versus 23 months (95% CI, 12-NR) (p=0.066) for patients with low and high miRNA-143 expression, respectively (**Figure 3**). A similar trend was reported for miR-206 expression. The group with low expression of miR-206 in tumor tissue showed a trend for longer DFS (mDFS=20 months vs 10 months, p=0.095) and OS (mOS=36 months vs 19 months, p=0.18) (**Figure 3**). No significant correlation was found for miR-155 expression and patients survival (**Figure 3**).

### 3.4 Univariate and multivariate analysis

In the entire cohort, Cox univariate analysis showed that lymph node involvement was significantly associated with DFS (p=0.016) and OS (p=0.03) and site of primary tumor was associated with OS (p=0.015). In the subset of patients with available data on miRNAs, miR-143 expression was significantly associated with DFS (p=0.007) and OS (p=0.01). Multivariate Cox analysis confirmed the association between miR-143 and OS (p=0.01). Other variables, including sex, age, miR-155, and miR-206 did not show a statistically significant effect on survival (**Tables 4A**–**D**).

**Table 4A:** Univariate Cox analysis for OS of the overall PC population (N=44).

OS			
N=44		Univariate	
Covariate		HR (95% CI)	p.value
Sex	Male (reference)		
	Female	1.1 (0.52-2.5)	0,75
Age		1 (0.96-1.1)	0,78
Site	Head (reference)		
	Body/Tail	0.78 (0.34-1.8)	0,56
pTMN	N0 (reference)		
	N+	4.7 (1.4-16)	0,03

The reference group is reported for each categorical covariate. Hazard ratios and confidence intervals are provided for each covariate.

**Table 4B:** Uni- and Multivariate Cox analysis for OS of the subset of PC patients with miRNA expression data N=25.

OS					
N=25		Univariate		Multivariate	
Covariate		HR (95% CI)	p.value	HR (95% CI)	p.value
Sex	Male (reference)				
	Female	1.2 (0.47-3.2)	0,68		
Age		1 (0.95-1.1)	0,73		
Site	Head (reference)				
	Body/Tail	0.48 (0.17-1.3)	0,15		
miR-155		1 (0.98-1)	0,92		
miR-143		1.1 (1-1.2)	0,01	1.1 (1-1.3)	0,01
miR-206		1 (0.99-1.1)	0,059	1 (1-1.1)	0,053

The reference group is reported for each categorical covariate. Hazard ratios and confidence intervals are provided for each covariate.

**Table 4C:** Univariate Cox analysis for DFS of the overall population N=44.

DFS			
N=44		Univariate	
Covariate		HR (95% CI)	p.value
Sex	Male (reference)		
	Female	1.3 (0.6-2.7)	0,51
Age		0.99 (0.95-1)	0,73
Site	Head (reference)		
	Body/Tail	1.1 (0.49-2.3)	0,88
pTMN	N0 (reference)		
	N+	3.7 (1.3-11)	0,016

The reference group is reported for each categorical covariate. Hazard ratios and confidence intervals are provided for each covariate.

**Table 4D:** Univariate Cox analysis for DFS of the subset of patients with miRNA expression data N=25. Bold = P<0.05.

DFS			
N=25		Univariate	
Covariate		HR (95% CI)	p.value
Sex	Male (reference)		
	Female	0.81 (0.32-2.1)	0,66
Age		0.97 (0.92-1)	0,23
Site	Head (reference)		
	Body/Tail	0.75 (0.28-2)	0,56
miR-155		1 (0.98-1)	0,95
miR-143		1.2 (1-1.3)	**0,007**
miR-206		1 (0.99-1.1)	0,16

The reference group is reported for each categorical covariate. Hazard ratios and confidence intervals are provided for each covariate.

bold= P<0.05.

### 3.5 TCGA analysis

**Figure 4:**
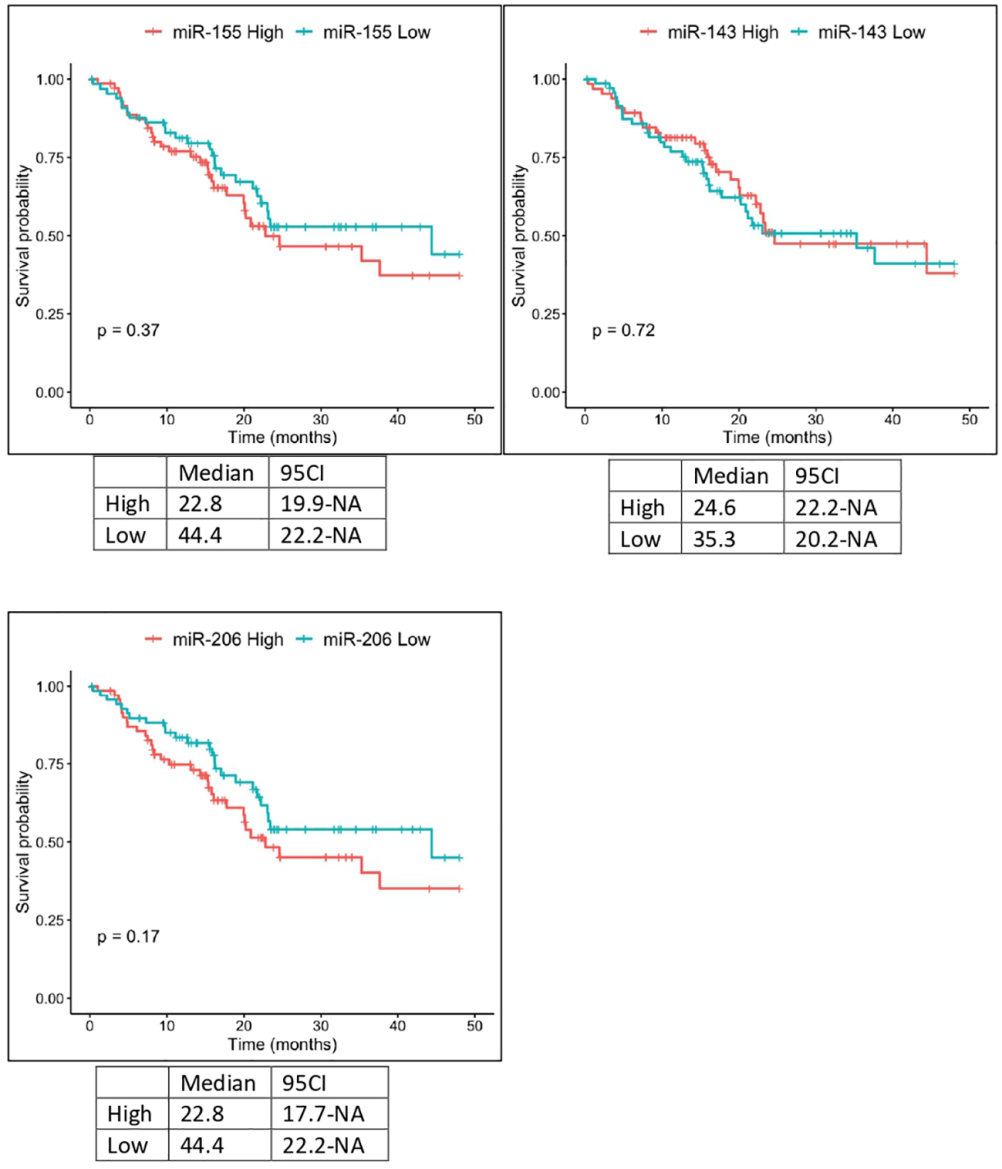
Kaplan-Meier curves for OS by miR-155, miR-206 and miR-143 expression from TCGA analysis. Significance levels have been computed with log-rank tests.

The expression of miR-143 in TCGA samples showed a statistically significant correlation with the anatomic neoplasm subdivision. Tumor samples expressing higher levels of miR-143 were predominantly localized within the pancreas head region, as opposed to the body and tail. Furthermore, TCGA patients with lower expression levels of miR-155 and miR-206 showed a higher OS, with median OS of 22.8 vs 44.4 months in both cases. These results, however, did not reach the threshold of statistical significance (**Figure 4**).

## 4 Discussion

The prognostic stratification of patients with resectable PC remains an unmet need. The current treatments provide only limited benefit and the curative rate is suboptimal. The identification of prognostic factors appears critical in order to tailor the optimal postoperative management of patients with resected PC (3, 21). KRAS mutations act as drivers for the vast majority of PCs (6). Therefore, we aimed to investigate the role of candidate miRNA involved in the RAS pathway. Although limited in sample size, our study prospectively enrolled a patient population highly representative of the routine clinical practice. As expected, the recurrence rate was 65.9% and this was consistent with the study of Conroy et al. where 3-year DFS rates were 39.7% in the mFOLFIRINOX arm and 21.4% in the gemcitabine arm. In addition, the PRODIGE 24 trial identified the type of adjuvant treatment regimen, age, grade, pathological staging, and center volume as significant prognostic factors for OS (22). Overall, there is a general consensus that regional lymph node involvement is one of the main prognostic factors in patients who underwent surgical resection for PC (23–25). Analyzing 1666 patients with resected PC from a large dataset, Schwarz et al. showed that pathological lymph node involvement was the factor that mainly influenced the survival (p<0.0001), with the most favorable prognosis observed for patients with 10-15 negative lymph nodes (24). In recent years, using a multivariable-adjusted model, Morales-Oyarvide et al. confirmed that regional nodal metastasis after surgical resection, also as assessed by AJCC 8th edition, was a reliable predictor of disease recurrence, DFS, and OS (25).

According to the literature, our case series confirmed pathological nodal involvement as one of the worst prognostic factors in patients with resected PC. Patients with pN+ stage had a reduced DFS (p=0.009) and OS (p=0.021) compared with those with pN0 stage. Interestingly, miR-143 was differentially expressed according to pN status: miR-143 was higher in patients with nodal involvement compared with those with pN0 and this results in a trend toward shorter DFS in patients with high miR-143 expression. In TCGA analysis, a statistically significant correlation was observed between miR-143 expression and primary tumor location (head vs body-tail) and this could contribute to the different course of the disease, with different prognostic implications. A higher frequency of recurrence in patients with high levels of miR-143 expression was also reported by Xu B et al. (26). Kent et al. revealed that miR-143/145 expression is repressed by activated KRAS, and the repression of miR-143 is crucial for the KRAS-mediated oncogenesis and progression (27).

In patients who experienced disease recurrence, among the clinical prognostic factors, some studies showed a different prognostic impact according to the specific site of metastasis. In detail, analyzing 13,233 patients with metastatic PC from a publicly available database, Oweira et al. showed that liver metastases were associated with shorter survival outcomes compared with liver-only (p<0.0001) or distant nodal-only metastases (p<0.0001) (28). The presence/absence of liver metastasis was also included as a stratification factor in the international, multicenter, open-label, randomized, phase 3 MPACT trial. Although the combination of nab-paclitaxel and gemcitabine offered a survival advantage compared with gemcitabine monotherapy in this subgroup (HR 0.69), the presence of liver metastasis was identified as an independent prognostic factors of OS and progression-free survival (PFS) (29, 30). Consistent with the literature, our study confirmed liver metastasis as the main negative prognostic factor in patients who experienced disease recurrence (p=0,027).

In our patient population, the expression of miR-155 was higher in tumor than in healthy tissue and this was consistent with the study by Wang et al. in which the upregulation of miR-155 was a consequence of the transitory or prolonged activation of KRAS oncogenic signal. Analyzing the downstream signaling pathway, they showed the fundamental role of MAPK and NF-kB as mediators between KRAS activation and miR-155 expression. Furthermore, the increased expression of miR-155 was associated with ROS accumulation and neoplastic growth of PC (31). Several observations correlate miR-155 expression with the JAK–STAT signaling pathway through the inhibition of SOCS1 and Ptpn2, suggesting its deep implication in inflammatory signal pathways and cancer (32, 33). Interestingly, the association between miR-155, tissue inflammation and carcinogenesis was also reported by Svrcek et al. in patients with inflammatory bowel disease-related colorectal cancer. In this population, an overexpression of miR-155 was found both in tumor tissue and in inflamed colonic mucosa of patients with inflammatory bowel disease (34).

Consistent with the study by Keklikoglou et al., we confirmed the downregulation of miR-206 in PC. miR-206 acts as a tumor suppressor in PC and was found to inhibit both the KRAS and ANXA2 oncogenes. Moreover, miR-206 is a negative key regulator of oncogenic KRAS-induced NF-κB transcriptional activity, which leads to reduced proangiogenic and proinflammatory factors that further result in tumor growth and poor prognosis. In PC cells, miR-206 overexpression was also associated with inhibition of VEGF-C in a largely unknown NF-κB-independent way. In addition, in a xenograft model, the authors showed that miR-206 was able to delay tumor growth (35). In our patient cohort, miR-206 high-expression level was associated with a numerically lower DFS. This result was confirmed by the trend towards a worse OS observed in the TCGA analysis.

Our study have several limitations, including the limited sample size that precluded a precise evaluation of the prognostic impact of candidate miRNAs, the lack of blinded pathological evaluation, and the heterogeneous disease assessments and post-surgical treatments.

Although limited in sample size, our study confirms the involvement of KRAS related miRNAs expression in tumorigenesis and metastatic spread in PC. As a perspective, a comprehensive analysis of the miRNA-RAS interplay could elucidate the impact of specific miRNAs on clinical outcome and prognostic stratification, especially for miR-143 overexpression which in our case series was associated with lymphatic spread and, consequently, poor prognosis.

## Data availability statement

The raw data supporting the conclusions of this article will be made available by the authors, without undue reservation.

## Ethics statement

The studies involving humans were approved by Comitato Etico Regionale for clinical experimentation of Toscana region (Italy), Area Vasta Centro section. The studies were conducted in accordance with the local legislation and institutional requirements. The participants provided their written informed consent to participate in this study.

## Author contributions

DL: Conceptualization, Data curation, Investigation, Writing – original draft, Writing – review & editing. SPo: Conceptualization, Data curation, Formal analysis, Investigation, Writing – original draft, Writing – review & editing. AT: Investigation, Writing – review & editing. FS: Data curation, Formal analysis, Investigation, Methodology, Software, Writing – original draft, Writing – review & editing. LMe: Formal analysis, Investigation, Writing – review & editing. EC: Data curation, Investigation, Writing – original draft, Writing – review & editing. LMo: Investigation, Writing – review & editing. AGu: Investigation, Writing – review & editing. GG: Investigation, Writing – review & editing. AGa: Conceptualization, Data curation, Formal analysis, Investigation, Supervision, Writing – review & editing. SPi: Conceptualization, Data curation, Formal analysis, Investigation, Methodology, Writing – original draft, Writing – review & editing. LA: Conceptualization, Investigation, Supervision, Writing – original draft, Writing – review & editing.
